# Hydrogen and formate production and utilisation in the rumen and the human colon

**DOI:** 10.1186/s42523-022-00174-z

**Published:** 2022-03-14

**Authors:** William J. Kelly, Roderick I. Mackie, Graeme T. Attwood, Peter H. Janssen, Tim A. McAllister, Sinead C. Leahy

**Affiliations:** 1 New Zealand Agricultural Greenhouse Gas Research Centre (NZAGRC), Palmerston North, New Zealand; 2 University of Illinois, Urbana-Champaign, USA; 3 AgResearch, Grasslands Research Centre, Palmerston North, New Zealand; 4 Agriculture and Agri-Food Canada, Lethbridge, Canada

**Keywords:** Hydrogen, Formate, Rumen, Colon, Methane, Methanogens, Homoacetogens, Mitigation

## Abstract

Molecular hydrogen (H_2_) and formate (HCOO^−^) are metabolic end products of many primary fermenters in the mammalian gut. Both play a vital role in fermentation where they are electron sinks for individual microbes in an anaerobic environment that lacks external electron acceptors. If H_2_ and/or formate accumulate within the gut ecosystem, the ability of primary fermenters to regenerate electron carriers may be inhibited and microbial metabolism and growth disrupted. Consequently, H_2_- and/or formate-consuming microbes such as methanogens and homoacetogens play a key role in maintaining the metabolic efficiency of primary fermenters. There is increasing interest in identifying approaches to manipulate mammalian gut environments for the benefit of the host and the environment. As H_2_ and formate are important mediators of interspecies interactions, an understanding of their production and utilisation could be a significant entry point for the development of successful interventions. Ruminant methane mitigation approaches are discussed as a model to help understand the fate of H_2_ and formate in gut systems.

## Introduction

Organoheterotrophic anaerobic microorganisms derive their energy by breaking down biomass and fermenting the monomers and oligomers that are released. In anoxic environments this fermentation is typically catalysed by syntrophic interactions between microorganisms. The anaerobic environments in mammalian gut ecosystems are microbial habitats that differ from non-gut systems in that they have relatively short digesta residence times with turnover once or twice per day, compared to anaerobic bioreactors (> 14–20 days) and sediments (years and decades). The faster turnover in gut systems results in incomplete fermentation with the generation of mainly volatile fatty acids (VFAs), methane (CH_4_), and carbon dioxide (CO_2_). There is also a need for relatively fast ATP generation so that microbes can grow fast enough so they are not washed out of the system. The VFAs are absorbed from the gut and serve as energy substrates and metabolites for the host animal while CH_4_ and CO_2_ are emitted as gaseous wastes [1]. In the foregut (rumen) of ruminants and the human large intestine CH_4_ and CO_2_ account for around 10 and 17% of the fermentable carbon respectively, with the remainder found as VFAs [2, 3]. In contrast, in biodigesters and sediments where VFAs are also metabolised, organic material is completely degraded to CH_4_ and CO_2_ [1].

In 1981 Meyer Wolin [3] reviewed what was then known about fermentation in the rumen and the human large intestine. This review highlighted the role of H_2_ and formate production and utilisation in these environments, and the importance of interactions between different microbes in interspecies H_2_ transfer. It also discussed approaches to reduce CH_4_ production in order to improve animal performance and feed utilisation. Since that time our knowledge of gut environments has rapidly increased. While the most significant development has been determining the diversity of microbial species in these gut environments, there have been advances in understanding the overall metabolism of the microbes and the enzymes encoded in their genomes. Besides interspecies H_2_ transfer, interspecies formate transfer has been realized to be of importance in mediating electron flow [4]. Also, it has been shown that the gut environment is not homogeneous but consists of planktonic and aggregated cell fractions (biofilms) associated with particulate matter and gut surfaces that have different passage rates and in which interspecies electron flow is significantly different [5, 6]. In recent years emphasis has been placed on research to understand the contribution of the gut microbiome to human health and to mitigate the environmental effects of ruminant CH_4_ emissions. However, there is still much to learn about how gut microbes interact with each other, with dietary components and with their mammalian host. In this review we examine mutualistic fermentative digestion in the ruminant anterior forestomach (rumen-reticulum or rumen) and the distal human colon emphasising the role that H_2_ and formate production and use play in their metabolisms. We also discuss progress in interventions that could potentially redirect H_2_ and formate metabolism and impact ruminant methane production.

## Differences between the two gut environments

The ruminant rumen is a large, pre-gastric fermentation organ in which mutualistic microbial fermentation takes place prior to gastric digestion. In contrast, the human colon is a much smaller, post-gastric fermentation chamber. Important features of these two gut fermentation systems are listed in Table 1. For studies of the human gut most samples are of faecal material or are obtained via other non-invasive techniques, whereas for ruminants collection of digesta directly from the rumen of cannulated animals or by oral intubation is well established [7, 8]. Furthermore, in ruminants the composition of the diet and the administration of feed additives can be precisely controlled making scientific experimentation stronger and more rigorous than the investigation of the human colon.

**Table 1:** Comparison of the rumen with the human colon—characteristics and properties of fermentative digestion in these gut compartments that specialize in fermentative digestion

Characteristic	Rumen	Human colon
Mode of digestion	Pregastric—foregut fermentor	Postgastric—hindgut fermentor
	Continuously stirred/mixed tank reactor	Plug flow tubular system
Diet	Evolved for efficient fibre degradation and utilisation. Major metabolizable energy supply (~ 70%) of the host energy requirements	Adapted to hydrolysing and fermenting undigested dietary residues and host endogenous secretions. Minor contribution to host energy requirements
	Breakdown dietary protein and non-protein nitrogen for synthesis of microbial protein	Post absorptive compartment
	Synthesis of B vitamins	Post absorptive compartment
	Rumen system evolved for detoxification/biotransformation of phytotoxins and mycotoxins	Host system evolved for transport and excretion of toxic/xenobiotic compounds
Blood glucose	Low—rely on gluconeogenesis to generate glucose precursors	High
Microbiology	Anaerobic bacteria and methanogenic archaea	Anaerobic bacteria and methanogenic archaea
	Ciliate rumen protozoa	Flagellate protozoa?
	Anaerobic rumen fungi	
	Bacteriophage	Bacteriophage
VFA/SCFA	Acetate, propionate and butyrate are the predominant volatile fatty acids	Similar molar proportions of the three main volatile fatty acids
	Branched chain VFAs	Similar
	Lactate → Propionate	Similar turnover
	Succinate → Propionate	Similar turnover
Gas composition	CO_2_ 65%; CH_4_ 27%; N_2_ 7%; O_2_ 0.6%; H_2_ 0.2%	CO_2_ 10%; CH_4_ 14%; N_2_ 65%; O_2_ 2.3%; H_2_ 3%
	CO_2_ produced from fermentation and HCO_3_^−^ in saliva. N_2_ and O_2_ is ingested with feed and diffuses through the rumen wall. Partial pressure of H_2_ maintained at a very low level. CH_4_ emission amounts to 2–12% of gross energy	N_2_, and O_2_ ingested, with CO_2_, H_2_ and CH_4_ resulting from colonic fermentation. Less CO_2_ and CH_4_ than the rumen but with higher H_2_ concentrations
Gas elimination	Mainly eructation	Flatus and reabsorption and removal by lungs

Both environments harbour dense and diverse populations of microorganisms that form closely integrated ecological units with their hosts. The rumen is inhabited by a diverse microbial community comprised of anaerobic bacteria, methanogenic archaea, ciliate protozoa, anaerobic phycomycete fungi and bacteriophage and is known to be highly adaptable metabolically to deal with changes in diet. One main difference of the rumen compared to the human colon is the presence of a large eukaryal population of ciliate protozoa that accounts for as much as 50% of the microbial biomass. Under normal healthy conditions, the human colon houses mainly anaerobic bacteria, methanogenic archaea, and bacteriophage. In both systems, the microbial inhabitants play vital roles in the nutritional, physiological, immunological, protective, and developmental functions of their respective hosts, but the forces that control and shape the composition and activities of these microbial communities remain poorly understood.

A major difference between the rumen and the colon is that in the rumen the microbes initiate feed degradation, while in the colon the host digestive processes act on the feed first. The diet of farmed ruminants is largely composed of fibre (cellulose, hemicelluloses, and pectin) and starch in varying proportions depending on the production system with a relatively constant daily intake. The human diet is highly variable and the fermentation substrates which reach the colon include undigested dietary polysaccharides such as fibre, resistant starch, and oligosaccharides that escape digestion in the upper tract. Host-secreted mucin glycans are also an important substrate for human gut microbes [9]. Rumen microbes are not thought to use host glycans, but the presence of host glycan-degrading enzymes in some rumen *Prevotella* spp. [10] suggests they may be able to use salivary glycoproteins.

Acetic, propionic, and butyric acids are the major VFA products of fermentation in both the rumen and human colon. It is well established that rumen VFAs are absorbed and contribute about 70% of the animals metabolizable energy requirement [11]. VFAs are also absorbed from the human large intestine and contribute to energy requirements of the host albeit at a much lower level (~ 10%, [11]). An important difference lies in the production of gaseous products. Intestinal gases of humans [12] have a lower percentage of CO_2_ and CH_4_ and a greater percentage of H_2_ than gases found in the rumen. CH_4_ emission is universal in rumen fermentation, whereas the proportion of humans identified as CH_4_ emitters varies [13, 14], with 20% of Western populations identified as high emitters [15]. Moreover, H_2_ is rarely a final product of rumen fermentation but is always a product of large intestinal fermentation in humans and significant amounts of residual H_2_ that is not used by microbes are excreted via expiration or flatus.

## Hydrogen and formate metabolism in the rumen

H_2_ is primarily produced during microbial fermentation by hydrogenases. These enzymes catalyse the reoxidation of cofactors reduced during carbohydrate fermentation [16] and dispose of the derived electrons by reducing protons to produce H_2_. In the rumen most of the H_2_ produced is used by methanogenic archaea to reduce CO_2_ (hydrogenotrophic methanogenesis) or methyl compounds (methylotrophic methanogenesis) to CH_4_, via a process known as interspecies hydrogen transfer [17]. H_2_ is maintained at sufficiently low concentrations through methanogenesis for fermentation to remain thermodynamically favourable [16, 18].

A range of rumen microbes belonging to several different phyla have been shown to produce H_2_, with 65% of cultured rumen bacterial and archaeal genomes [10] encoding enzymes that catalyse H_2_ production or consumption [19]. Metagenome assembled genomes (MAGs) from different gastrointestinal tract regions of seven ruminant species [20] generated similar results. A total of 6,152 [NiFe]-, [FeFe]-, and Fe-hydrogenase-containing MAGs were detected, 3003 of which encoded enzymes for fermentative H_2_ production (72.7% from the Firmicutes), while 95 MAGs encoded H_2_-uptake hydrogenases and the methyl-CoM reductases related to hydrogenotrophic methanogenesis (mainly from *Methanobrevibacter*).

Flavin-based electron bifurcation is an electron pair-splitting mechanism that enables the coupling of energy-producing redox reactions with energy-consuming electron transfer reactions [21] and is likely to be particularly important for fermentation in the anaerobic gut environment. Metatranscriptomic analysis using data from sheep that differed in their methane yield [22] showed that electron-bifurcating [FeFe]-hydrogenases were key mediators of ruminal H_2_ production [19]. Hydrogenases from carbohydrate-fermenting Clostridia (*Ruminococcus*, *Christensenellaceae* R-7 group) accounted for half of all hydrogenase transcripts, suggesting that these organisms generate much of the H_2_ used by the hydrogenotrophic *Methanobrevibacter* species. Co-culturing experiments showed that the hydrogenogenic cellulose fermenter *Ruminococcus albus* expressed its electron-bifurcating hydrogenase and suppressed its ferredoxin-only hydrogenase when grown with the hydrogenotrophic fumarate reducer *Wolinella succinogenes* [19, 23].

Rumen methanogens also participate in symbiotic relationships with protozoa that produce large quantities of H_2_ via their hydrogenosomes [24]. In return, the protozoa benefit from H_2_ removal as high H_2_ partial pressure is inhibitory to their metabolism. Meta-analysis of protozoa defaunation studies concluded that elimination of ciliate protozoa reduced CH_4_ production by up to 11% [25]. A similar relationship exists between methanogens and anaerobic rumen fungi which also contain hydrogenosomes [26].

Metatranscriptomic studies [19] showed that, while enzymes mediating fermentative H_2_ production were expressed at similar levels, methanogenesis-related transcripts predominated in high methane yield sheep, while alternative H_2_ uptake pathways were significantly upregulated in low methane yield sheep. These other H_2_ uptake pathways could potentially limit CH_4_ production by redirecting H_2_ uptake away from methanogenesis towards homoacetogenesis (*Blautia*, *Eubacterium*), fumarate and nitrite reduction (*Selenomonas*, *Wolinella*), and sulfate reduction (*Desulfovibrio*). Homoacetogens produce acetate from H_2_ and CO_2_ and are known to occur in the rumen, but their abundance is generally lower than hydrogenotrophic methanogens [27, 28]. It is likely that methanogens outcompete homoacetogens at the low H_2_ concentrations in the rumen [29]. Nitrate and sulfate reduction are thermodynamically more favourable than methanogenesis and homoacetogenesis [16], and nitrate and sulfate-reducing bacteria occur naturally in the rumen. Their population densities increase as the concentration of their respective electron acceptors in the ruminant diet increases. However, nitrate and sulfate concentrations in ruminant diets are usually very low so these processes would be substrate limited and their end products can be toxic at high concentrations [30].

Much less is known about formate concentrations in the rumen or the significance of formate as an electron carrier between species [31]. At relatively high redox potentials (low H_2_ and formate concentrations), formate is thermodynamically and kinetically a more favourable interspecies electron carrier than H_2._ This is of importance mainly for planktonic microbes, where the distances for electron transfer between organisms are greater than between those growing in biofilms and aggregates [5]. Many rumen microbes contain formate dehydrogenase genes, but their expression under different conditions has been much less studied compared to hydrogenase genes.

## Hydrogen and formate metabolism in the human colon

Less is understood about the H_2_ and formate economy of the human gastrointestinal tract. H_2_ is formed in large volumes in the colon as an end product of polymeric carbohydrate fermentation, for example by members of the Firmicutes and Bacteroidetes. Two major pathways for disposal of H_2_ are known, methanogenesis, and homoacetogenesis, with homoacetogenesis being the predominant pathway [32, 33]. When sulfate is available, dissimilatory sulfate reduction is carried out by sulfate-reducing bacteria but in its absence these bacteria thrive by producing H_2_ rather than oxidizing it. Most methanogens require H_2_ or formate for growth whereas homoacetogens are metabolically versatile and can also utilise a wide range of organic substrates. The dominance of one pathway over another appears to vary among individuals and to be inherited; there are individuals in which most of the H_2_ generated is converted to CH_4_ and in others H_2_ can accumulate to high concentrations [12]. Spatial and temporal variations in the chemical composition of the digesta in the human colon could result in specific microhabitats that support different H_2_ and/or formate metabolizing microbes. Homoacetogenesis is energetically less favourable than methanogenesis but may offer greater benefits to the host as CO_2_ and H_2_ are converted to acetate for use as an energy source, with no evolution of gas. Microbes using these two pathways represent keystone species in the human gut microbiome [34].

Although the formation and use of H_2_ and/or formate in some of the colonic microbes are well studied in pure culture, the hydrogenases and formate dehydrogenases responsible have not been investigated and our understanding of their roles at different stages of the fermentation process is poor. Only one study has surveyed the genomic and metagenomic distribution of hydrogenase-encoding genes in the human colon to infer the dominant mechanisms of H_2_ cycling [35]. Most microbial species from the Human Microbiome Project Gastrointestinal Tract reference genome database encoded the genetic capacity to form or use H_2_. A wide variety of anaerobically adapted hydrogenases were present, with [FeFe]-hydrogenases predominant. Metagenomic analysis of stool samples from 20 healthy humans indicated that the hydrogenase gene content of all samples was overwhelmingly dominated by fermentative and electron-bifurcating [FeFe]-hydrogenases from members of the Bacteroidetes and clostridial members of the Firmicutes. This study concluded that H_2_ metabolism in the human colon is driven by fermentative H_2_ production and interspecies H_2_ transfer using electron-bifurcation rather than respiration as the dominant mechanism of H_2_ reoxidation. However, microenvironmental niches in this mucosal ecosystem have not been fully studied and both the cellular and genetic bases for H_2_ and formate cycling in the human colon require further exploration.

Bacteria belonging to the family *Christensenellaceae* are highly heritable members of the human gut microbiome that show strong correlations with host health [36]. Analysis of human gut metagenomes have identified positive associations between *Christensenella* spp. and *Methanobrevibacter smithii*. In co-cultures these organisms grow together in dense flocs, and H_2_ generated by *Christensenella* spp. supports CH_4_ production by *M. smithii* [37]. This interaction shifts fermentation end products toward more acetate and less butyrate, potentially affecting the physiology of the human host. Interaction between *M. smithii* and members of the *Christensenellaceae* R-7 group has also been highlighted in analysis of the microbiomes of 30 subjects identified as high or low CH_4_ emitters [15]. High emitters were characterised by a 1000-fold increase in *M. smithii* which co-occurred with bacteria from a core group of keystone species from the *Christensenellaceae* and *Ruminococcaceae* families*.* Cross feeding between H_2_ and formate-producing bacteria and the human gut homoacetogen *Blautia hydrogenotrophica* has also been demonstrated in co-cultures [38–40].

## Redirecting hydrogen metabolism and ruminant methane mitigation

While there is interest in trying to determine if a mechanistic/causative relationship exists between the presence of methanogens and human gut health [15, 41], there has been increasing urgency in developing approaches that can practically mitigate CH_4_ from ruminant animals [42]. Globally, CH_4_ from enteric fermentation in ruminant livestock is a major source of agricultural greenhouse gases [43, 44]. Ideally, any developed mitigation approach should induce a co-benefit for the animal, for example enhanced production or health. Co-benefits can help drive practical adoption of the technology on farm. While research into reducing ruminant CH_4_ emissions has been in progress for many years [45, 46] and promising mitigation approaches are being developed [47], emergence of co-benefits from these approaches is not being observed consistently. This is contrary to the frequently stated hypothesis that ruminal CH_4_ production represents a loss of energy, from 2 to 12% of gross energy intake [48], which could in principle otherwise be available for animal growth or milk production. Historically, it has also been hypothesized that H_2_ accumulation resulting from the inhibition of methanogenesis will impair fibre digestion and fermentation [18]. It is becoming clear that a lack of understanding of H_2_ and formate metabolism and how it can be manipulated is a barrier that needs to be overcome in order to support the development of CH_4_ mitigation approaches with co-benefits. Emerging CH_4_ mitigation strategies in ruminants are now available and can provide model systems to help advance our knowledge in this area. Four promising areas are discussed below.

### Animal selection and breeding

Animals vary in their methane production and breeding low methane emitting animals is one mitigation approach. Significant progress has been made with sheep where studies have found animals that vary naturally in the amount of CH_4_ they produce. The heritability of this trait has enabled the breeding of low-CH_4_ emitting sheep [49, 50], and CH_4_ emissions from selected, divergent lines differ on average by 10–12%. Physiological characteristics such as a reduction in the rumen retention time of feed particles [51] and reduced rumen volume [52] are factors likely to contribute to the low CH_4_ emissions. There are also differences observed in their rumen microbial communities [53] and expression of microbial genes involved in the production of CH_4_ are reduced in low-CH_4_ sheep [22]. Kamke et al. [54] proposed that the rumen microbiome in low-CH_4_ animals supported heterofermentative growth leading to lactate production, with the lactate subsequently metabolised mainly to butyrate. Greening et al. [19] offered an alternative interpretation with H_2_ uptake through non-methanogenic pathways accounting for the differences observed.

### Competing terminal electron acceptors or alternative H_2_ users

Several alternative electron acceptors have been added to ruminant diets in attempts to alter the rumen fermentation and reduce CH_4_ production. Nitrate is the most studied compound [46] and is reduced via nitrite to ammonia, reducing the availability of H_2_ for CH_4_ synthesis. Sulfate reduction will also compete for electrons and H_2_ and may lower CH_4_ production [30]. Stimulating the activity of acetogens through the inhibition of methanogens has been proposed as a strategy for ruminant CH_4_ mitigation [55], but it is unclear whether resident rumen homoacetogens could fulfil the H_2_ disposal role or whether non-resident homoacetogens would need to be inoculated into the rumen [56]. Studies to date where methanogenesis has been inhibited with an effective methane inhibitor, such as 3-nitrooxypropanol, have not demonstrated increased homoacetogenesis.

In the gut of macropod marsupials, which consume a diet similar to ruminants, CO_2_ is mainly reduced to acetate rather than to CH_4_ as a means of electron disposal [28]. The reason for the preference of this alternative pathway in macropods is unknown, but it has been argued that their tubiform forestomach lacks the mechanisms to remove gaseous products of fermentation (e.g., eructation in the rumen and flatus in the lower bowel) and their immune secretions suppress the microbes responsible for releasing H_2_ or CH_4_ to prevent gas pressure build-up that would threaten gut integrity [57].

### Methanogen inhibiting technologies

Several different approaches have been used to specifically target methanogens in the rumen. These include feed additives such as 3-nitrooxypropanol [58], halogenated compounds [59], and certain seaweeds [60] as well as work to develop anti-methanogen vaccines [61]. Studies to date with the available technologies suggest that when CH_4_ production is inhibited, we do not observe a sufficient increase in rumen H_2_ emissions to account for the reducing equivalents that are not captured in CH_4_. It is assumed that the electrons are being diverted to other fermentation products, such as acetate, propionate, butyrate, and microbial biomass, but the balance of this redirection of electrons is not well understood. The use of methanogen inhibitors in combination with microbes that could potentially redirect H_2_ to other products has yet to be explored.

An alternative approach is to target the microbes that produce the substrates for methanogenesis. Although recent work has begun to identify the bacteria most likely to produce H_2_ [19] and methyl compounds [62] used as substrates for methanogenesis in the rumen, significant knowledge gaps remain. At this point it is unknown if a reduction in the production of substrates for methanogenesis would impede overall fermentation.

### Diet

Although methane emissions arising from an individual animal are primarily driven by the quantity of feed eaten [63], the chemical composition of feeds can also influence emissions. Consequently, the nature of the feed consumed may select for microbial populations with different fermentation pathways that yield less H_2_ and therefore less CH_4_. For example, concentrate-based diets are associated with lower CH_4_ yield (g/kg DMI; [48]) because fermentation of starch in concentrate results in more propionate and butyrate being produced and less CH_4_. Fermentation products [64] and microbial composition [65] may be influenced by the oxidation state of the carbon substrates especially those with higher levels of the more reduced sugar alcohols or more oxidised sugar acids. Brassica forages have also been shown to result in lower CH_4_ yields in lambs than perennial ryegrass [66]. The reason for this is not understood but an altered rumen microbiota or the presence of bioactive glucosinolates in brassicas have been suggested as possible causes [67]. Generally, however, it takes large changes in diet to bring about significant changes in enteric methane emissions in ruminants.

## Conclusion

Here we have contrasted the rumen and human colon environments and H_2_ and formate have emerged as key metabolites involved in cross-feeding between members of the microbiota in both gut ecosystems with important roles in shaping the syntrophic networks that operate in these environments. The role of H_2_ and formate production as electron sinks for individual microbes and their transfer of electrons to homoacetogenic bacteria and methanogenic archaea are key functions to ensure ongoing polysaccharide degradation and energy generation for both ruminants and humans. Although the anaerobic bacteria and archaea are broadly similar in each environment, H_2_ produced in the rumen is consumed predominantly by incorporation into CH_4_, whereas in the human colon significant H_2_ emissions escape the system. Determining what controls these differences will be important in understanding the impact of H_2_ and formate turnover on human gut metabolism and in reducing the environmental impact of ruminant CH_4_. Currently, the availability of emerging CH_4_ mitigation approaches for ruminant animals makes the rumen an ideal gut system to study the production and utilisation of hydrogen and formate in gut systems and generate knowledge applicable to both systems.

Our present knowledge of the mammalian gut H_2_ and formate economy is incomplete and an improved understanding of the active groups of microbes involved in H_2,_ and formate metabolism is required. Cultures and genome sequences of model hydrogenotrophs, such as *Methanobrevibacter*, and *Blautia*, are available and have been used to demonstrate interspecies H_2_ and formate transfer in co-culture. However, our knowledge of which organisms produce the bulk of the H_2_ and formate in gut environments is limited. The metagenome- and metatranscriptome-based studies have highlighted the diversity of gut microbes encoding the signature genes for hydrogenotrophy and have emphasized the need for more exact information about the function of these genes in the gut environment. There also remains a need to bring a greater proportion of representatives of the currently uncultured microorganisms into cultivation together with additional host-associated homoacetogen and methanogen strains. This should be accompanied by studies of their physiology, metabolism, and interactions with other gut anaerobes to provide a body of knowledge beyond what can be inferred from genome and metagenome sequence data. With the increased availability of such pure cultures and their corresponding genome sequences it will prove possible to construct metabolically interacting microbial consortia so that the contributions of different microbes to overall community function can be ascertained.

## Data Availability

Not applicable.

## Abbreviations

H_2_: Hydrogen
HCOO^−^: Formate
VFAs: Volatile fatty acids
CH_4_: Methane
CO_2_: Carbon dioxide
MAGs: Metagenome assembled genomes
DMI: Dry matter intake
